# Cyclosporine A-Sensitive, Cyclophilin B-Dependent Endoplasmic Reticulum-Associated Degradation

**DOI:** 10.1371/journal.pone.0013008

**Published:** 2010-09-28

**Authors:** Riccardo Bernasconi, Tatiana Soldà, Carmela Galli, Thomas Pertel, Jeremy Luban, Maurizio Molinari

**Affiliations:** 1 Institute for Research in Biomedicine, Bellinzona, Switzerland; 2 Department of Microbiology and Molecular Medicine, University of Geneva, Geneva, Switzerland; 3 Ecole Polytechnique Fédérale de Lausanne, School of Life Sciences, Lausanne, Switzerland; Fred Hutchinson Cancer Research Center, United States of America

## Abstract

Peptidyl-prolyl *cis/trans* isomerases (PPIs) catalyze *cis/trans* isomerization of peptide bonds preceding proline residues. The involvement of PPI family members in protein refolding has been established in test tube experiments. Surprisingly, however, no data is available on the involvement of endoplasmic reticulum (ER)-resident members of the PPI family in protein folding, quality control or disposal in the living cell. Here we report that the immunosuppressive drug cyclosporine A (CsA) selectively inhibits the degradation of a subset of misfolded proteins generated in the ER. We identify cyclophilin B (CyPB) as the ER-resident target of CsA that catalytically enhances disposal from the ER of ERAD-L_S_ substrates containing *cis* proline residues. Our manuscript presents the first evidence for enzymatic involvement of a PPI in protein quality control in the ER of living cells.

## Introduction

Formation and reduction of covalent bonds between cysteine side chains and *cis/trans* isomerization of peptide bonds preceding proline residues are rate-determining steps for the attainment of the native and functional 3D structure of polypeptides synthesized in the ER. These reactions might also be rate-limiting for the unfolding of aberrant polypeptides that require retro-translocation (dislocation) across the ER membrane for proteasomal degradation [1]. *In vitro*, these reactions are catalyzed by protein disulfide isomerases (PDIs [2]) and by PPIs [3], [4], respectively. Extensive experimental evidence has shown the importance of PDIs-assisted polypeptide folding and unfolding in living cells [5], [6]. Despite 25 years of PPI catalysis experiments *in vitro*, a direct involvement of PPIs in catalysis of protein folding, in regulation of protein quality control or in clearance of misfolded polypeptides from the ER of living cells remains to be demonstrated [7], [8], [9].

Most polypeptides entering the ER lumen are covalently modified at asparagine side chains with glucose_3_-mannose_9_-N-acetylglucosamine_2_- oligosaccharides. Their maturation is assisted by a dedicated folding machinery comprising the oligosaccharide-binding chaperones calnexin and calreticulin and the oxidoreductase ERp57 [10]. Processing of oligosaccharides displayed on misfolded conformers by ER-resident α1,2-mannosidases, with removal of up to 4 terminal mannose residues, irreversibly extracts folding-defective polypeptides from the lectin-operated folding machinery [11]. In mammalian cells, two *ER-associated degradation (ERAD) shuttles*, OS-9 and XTP3-B [12], [13], [14], transport ERAD-L_S_ substrates (i.e. soluble, extensively de-mannosylated terminally misfolded glycopolypeptides) from the ER lumen to the site of dislocation across the ER membrane [15]. OS-9 and XTP3-B deliver ERAD-LS substrates to a multi-protein complex comprising the membrane receptor SEL1L, the associated E3 ubiquitin ligase HRD1 and an elusive dislocation (retro-translocation) channel [16]. The stringent requirement for HRD1, SEL1L and OS-9/XTP3-B for disposal is bypassed when the same misfolded domains are tethered to the ER membrane (ERAD-L_m_ substrates) [15], [17]. Thus, luminal misfolded polypeptides and membrane-tethered polypeptides with structural defects in the ER lumen have different requirements for efficient clearance from the ER.

Although the process of dislocation across the ER membrane is poorly defined, unfolding of aberrant polypeptide chains [18] and disassembly of disulfide-bonded protein aggregates [19] have been shown to facilitate protein clearance from the ER lumen. A role in ERAD has been demonstrated for several members of the PDI superfamily (e.g. PDI, ERp57, ERp72, ERp29, ERdj5), thus implying that reduction of inter- and intra-molecular disulfide bonds plays a crucial role in ERAD by eliminating tertiary and quaternary structures that could impair transport across a putative proteinaceous membrane dislocon (reviewed in [5]). On the same line, it is conceivable that the PPIs-catalyzed interconversion of *cis* into *trans* peptidyl-prolyl bonds could facilitate dislocation of ERAD substrates across the ER membrane by eliminating turns in the polypeptide secondary structure [9].

Here we report that the immunosuppressive drug CsA, a specific inhibitor of the cyclophilin family of PPIs, selectively delays the degradation of the ERAD-L_S_ substrate BACE457Δ leaving unaffected disposal from the ER of the same polypeptide when tethered to the ER membrane (the ERAD-L_M_ protein BACE457). This identifies CsA as the first inhibitor that selectively acts upon an ERAD-L_S_ substrate and not upon the corresponding ERAD-L_M_ polypeptide. We then extend this finding by showing that, among roughly 20 mammalian cyclophilin family members, CyPB is unique because it plays a crucial role in ERAD that requires its enzymatic activity. Importantly, CsA is not a general inhibitor of the ERAD-L_S_ pathway and CyPB is not required for disposal of all ERAD-L_S_ substrates. Rather, the presence of peptidyl-prolyl bonds in the *cis* conformation renders disposal of ERAD-L_S_ substrates sensitive to CsA and dependent on CyPB intervention. Altogether, our manuscript presents the first evidence for the enzymatic involvement of a PPI in protein quality control in the ER of a living cell.

## Results and Discussion

### CsA selectively inhibits disposal of BACE457Δ

BACE457 and BACE457Δ are splice variants of the human beta-site amyloid precursor protein cleaving enzyme BACE501 [20], an aspartic protease involved in generation of the Aβ peptide that forms plaques in the brain of Alzheimer's disease patients. A 44-residue deletion in the ectodomain prevents attainment of the native structure and results in degradation from the ER lumen when the proteins are ectopically expressed in cultured cells. Proteasome-dependent disposal of both proteins requires intervention of EDEM variants and extensive de-mannosylation of the 2 protein-bound N-glycans [19], [21], [22], [23]. However, degradation of BACE457Δ, an ERAD-L_S_ protein, strictly depends on HRD1, SEL1L and OS-9/XTP3-B, while disposal of BACE457, an ERAD-L_M_ protein, progresses efficiently even upon inactivation of the HRD1 pathway [15].

BACE457 and BACE457Δ contain 26 and 25 proline residues, respectively. It is impossible to establish if, and which one of the peptidyl bonds preceding these proline residues is converted from the trans to the *cis* configuration during the short retention of these folding-defective polypeptides in the ER lumen. It is of interest, however, that in the folding competent variant BACE501 the peptidyl bonds preceding Pro84, Pro146 and Pro390 are in the *cis* configuration (see below and Materials and Methods). To assess whether prolyl isomerases might facilitate disposal of BACE457 and BACE457Δ from the mammalian ER, we exposed cells transiently transfected for expression of either one of the two model substrates to CsA, a selective inhibitor of immunophilin members of the PPIs family [24]. CsA-treatment was compared with cell exposure to a series of well-characterized ERAD inhibitors (thapsigargin (Tg, which inhibits the SERCA pump thus depleting luminal calcium [25]); kifunensine (Kif, an inhibitor of α1,2-mannosidases [26]); PS341 (a proteasome inhibitor [27])).

**Figure 1:**
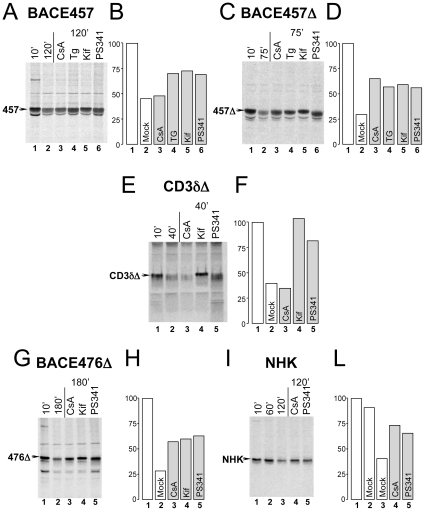
Consequences of CsA-treatment on disposal of ERAD-L_M_ and ERAD-L_S_ substrates. **A** Labeled BACE457, an ERAD-L_M_ substrate, was immunoisolated from total cell extracts with a specific antibody after 10 (lane 1, initial amount) or 120 min of chase (lane 2, residual amount). Labeled BACE457 was also immunoisolated from cells exposed for 120 min to CsA (lane 3), Tg (lane 4), Kif (lane 5) or PS341 (lane 6). **B** Quantification of the labeled polypeptide bands shown in the gel. Reproducibility of these data (i.e. lack of CsA inhibition) is confirmed by the independent experiment shown in **Figs. 2B–2C**, lanes 1–3. **C** Same as **A** for BACE457Δ, an ERAD-L_S_ substrate. Chase times are 10 (initial) and 75 min (residual). The apparent mass of BACE457Δ is reduced by the progressive and extensive de-mannosylation of the protein-bound oligosaccharides during the chase (lane 1 *vs* 2 [19], [35], [36]). Consistently, enhancement in electrophoretic mobility is specifically inhibited by Kif (lane 5 in **Figs. 1A** and **1C**; lane 4 in **Figs. 1E** and **1G** [26]). CsA, Tg and PS341 inhibit BACE457Δ disposal without affecting the enhancement of electrophoretic mobility during the chase. Thus, they all affect events occurring after substrate de-mannosylation. **D** Same as **B** for BACE457Δ. The reproducibility is confirmed in **Figs. 2D–2E**, lanes 1–3. **E** Same as **A** for CD3δΔ, an ERAD-L_S_ substrate lacking *cis* peptidyl-prolyl bonds. **F** Same as **B** for CD3δΔ. The reproducibility is confirmed in **Figs. 2F–2G**, lanes 1–3. **G** Same as **A** for BACE476Δ. **H** Same as **B** for BACE476Δ. **I** Same as **A** for NHK. **L** Same as **B** for NHK.

Seventeen hours after cell transfection, the ectopically expressed ERAD substrates were metabolically labeled for 10 min by incubating cells in a media containing ^35^S-methionine and -cysteine. The initial amount of labeled BACE457 (**Figs. 1A–1B**) or BACE457Δ (**Figs. 1C–1D**) was immunoisolated from cell lysates prepared after 10 min of chase in the absence of radioactivity (lane 1). To monitor ERAD, the residual amount of labeled BACE457 or BACE457Δ was immunoisolated after 120 or 75 min of chase, respectively, from mock-treated cells (lane 2) or from cells exposed to CsA, Tg, Kif or PS341 (lanes 3–6).

**Figure 2:**
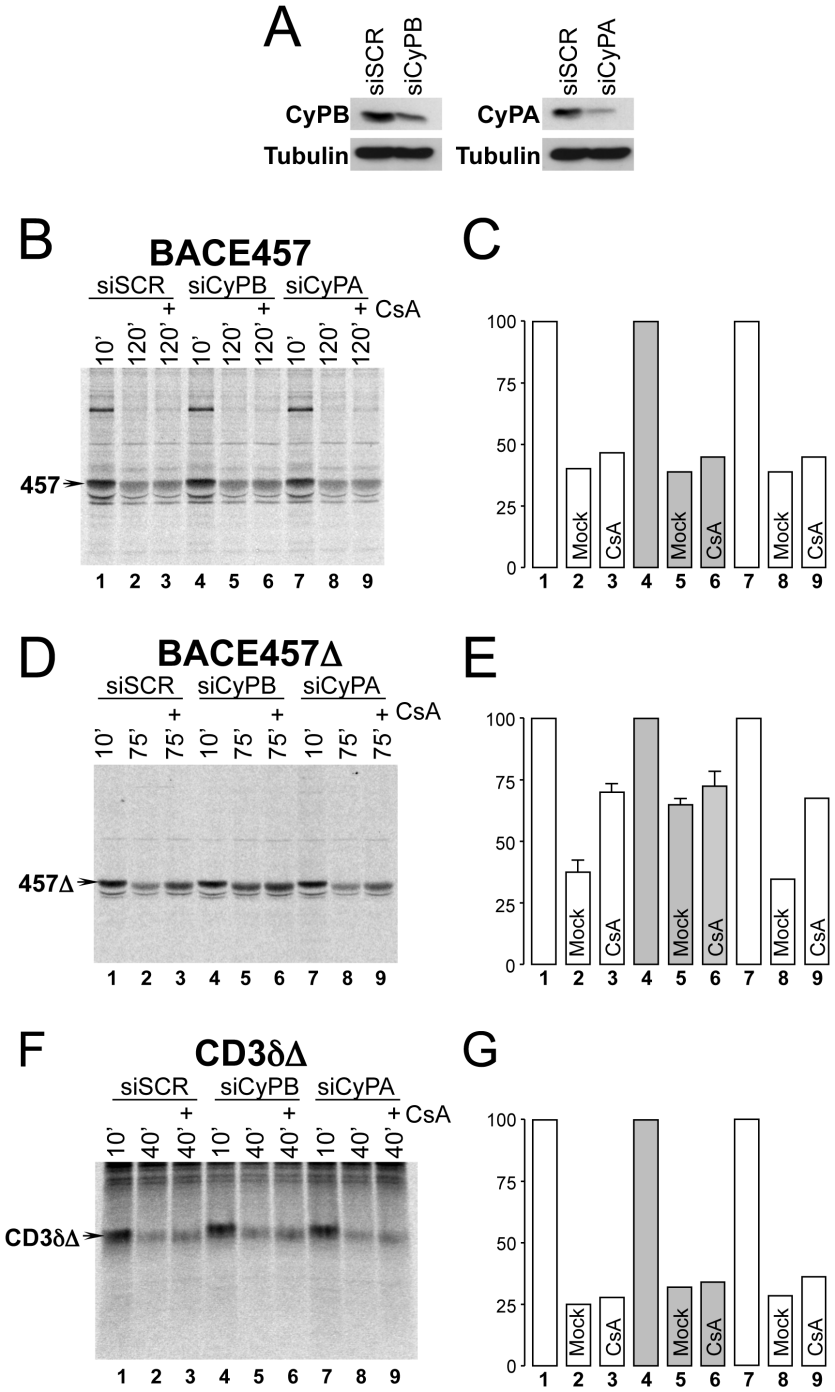
Consequences of CyPB or CyPA down regulation on BACE457, BACE457Δ and CD3δΔ disposal from the ER. **A** Down-regulations of CyPB and of CyPA were assessed by immunoblot of total cell lysates. Tubulin is a loading control. **B** Radiolabeled BACE457 was immunoisolated at the end of the chase times from detergent-extracts of cells expressing a scrambled siRNA (siSCR, lanes 1–3), a siRNA targeting CyPB (siCyPB, lanes 4–6) or CyPA (siCyPA, lanes 7–9) and exposed to CsA (lanes 3, 6 and 9). **C** Quantification of the labeled polypeptide bands. **D** Same as **B** for BACE457Δ. **E** same as **C** for BACE457Δ. Error bars represent SD from the mean of at least three independent experiments. **F** Same as **B** for CD3δΔ. **G** Same as **C** for CD3δΔ.

**Figure 3:**
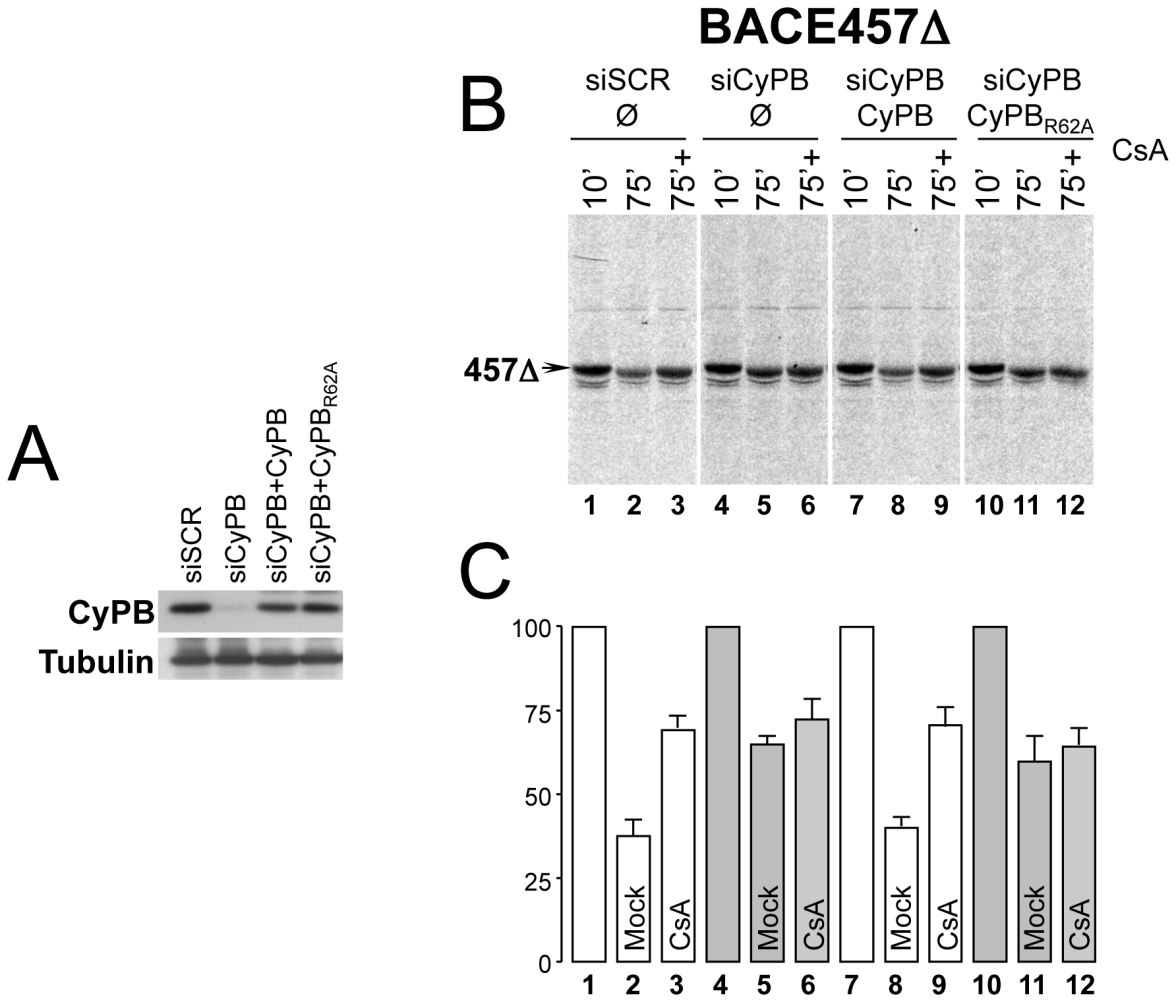
Reversibility of the ERAD defect requires back-transfection of enzymatically active CyPB. **A** Down-regulation of CyPB and back-transfections of active or catalytically inactive (R62A) CyPB were assessed by immunoblot of total cell lysates. Tubulin is a loading control. **B** Radiolabeled BACE457Δ was immunoisolated at the end of the chase times from detergent-extracts of cells expressing normal levels of CyPB (siSCR, lanes 1–3), in cells with reduced level of CyPB (siCyPB, lanes 4–6), in cells with reduced level of CyPB back-transfected with active (siCyPB+CyPB, lanes 7–9) or catalytically inactive CyPB (siCyPB+CyPB_R62A_, lanes 10–12). **C** Quantification of the labeled polypeptide bands. Error bars represent SD from at least two independent experiments.

Confirming published data [19], [21], [22], [23], Tg, Kif and PS341 substantially delayed disposal of both BACE457 and BACE457Δ (**Figs. 1A–1D**). CsA did not inhibit degradation of BACE457 (**Figs. 1A–1B**, lane 3 *vs* lane 2 and **Figs 2B–2C**, lanes 1–3), but substantially delayed the clearance from the ER lumen of BACE457Δ (**Figs. 1C–1D**, lane 3 *vs* lane 2 and **Figs 2D–2E**, **3B–3C**, lanes 1–3) as efficiently as the conventional ERAD inhibitors Tg, Kif and PS341 (**Figs. 1C–1D**, lanes 4–6). To summarize, we identify CsA as the first compound that selectively inhibits disposal of a soluble (ERAD-L_S_), but not of a membrane-tethered (ERAD-L_M_) variant of a misfolded polypeptide.

### CsA is not a general inhibitor of the ERAD-L_S_ pathway

To determine whether CsA is a general inhibitor of the ERAD-L_S_ pathway, we next checked whether cell exposure to CsA delayed disposal of CD3δΔ. Like BACE457Δ, this tri-glycosylated, soluble and folding-defective ERAD-L_S_ protein stringently depends on HRD1, SEL1L and OS-9/XTP3-B for efficient disposal [15]. As expected for an ERAD substrate, disposal of CD3δΔ was substantially delayed upon inactivation of protein de-mannosylation and upon inactivation of 26S proteasomes (**Figs. 1E–1F**, lanes 4 and 5, respectively). However, CsA was ineffective in preventing clearance of CD3δΔ from the ER (lane 3). Thus, even though CsA substantially delayed disposal of the ERAD-L_S_ protein BACE457Δ (and of other canonical ERAD-L_S_ substrates such as BACE476Δ (**Figs. 1G–1H**) and NHK, a folding-defective version of the secretory protein α1-antitrypsin (**Figs. 1I–1L**)), the incapacity of CsA to prevent CD3δΔ disposal showed that CsA is not a general inhibitor of the ERAD-L_S_ pathway.

Why is the disposal of CD3δΔ insensitive to CsA and the disposal of other ERAD-L_S_ substrates efficiently delayed by this PPI inhibitor? It is possible that none of the peptidyl-prolyl bonds of the misfolded CD3δΔ retained in the ER lumen is in the *cis* configuration, while one or more peptidyl-prolyl bonds of the misfolded BACE variants are in *cis* and must be isomerized to the *trans* conformation to promote efficient clearance from the ER. Of some relevance in this context could be that the corresponding native proteins do not have (the CD3δ in the functional T cell receptor) or do have peptidyl-prolyl bonds in the *cis* conformation (the native BACE501, Materials and Methods). We therefore hypothesized that the presence of peptidyl-prolyl bonds in the *cis* conformation determines CsA-sensitivity for the disposal of ERAD-L_S_ polypeptides from the mammalian ER (see next sections).

### CyPB is the luminal CsA target involved in ERAD

CsA is a cyclic undecapeptide produced by the fungus *Tolypocladium inflatum gams*. It is used in the clinic as an immunosuppressant to reduce the risk of graft rejection upon allogenic transplant and to improve short-term allograft survival [28]. The PPI family member CyPB is the ER-resident target of CsA [29]. A role for CyPB (or of any other PPI family member) in catalysis of peptidyl-prolyl *cis/trans* isomerization in protein biogenesis and/or quality control in the ER of living cells is not supported by experimental data. To determine whether CyPB intervenes in protein disposal from the ER lumen, we compared degradation of the ERAD-L_M_, CsA-insensitive substrate BACE457 (**Figs. 2B–2C**) and of the ERAD-L_S_, CsA-sensitive substrate BACE457Δ (**Figs. 2D–2E**) in cells with normal level of CyPB (lanes 1–3), with reduced level of CyPB (lanes 4–6) or with reduced level of CyPA, a cytosolic target of CsA (lanes 7–9). Down-regulation of the target proteins upon specific RNA interference is shown in **Fig. 2A**. The data shown in **Figs. 2B–2C** confirmed that cell exposure to CsA does not significantly delay disposal of the membrane-tethered BACE457 from the ER lumen (compare lane 2 with 3). Down-regulation of CyPB (**Figs. 2B–2C**, lanes 4–6) or of CyPA (lanes 7–9) had no significant consequences on BACE457 disposal. Thus, CyPB is dispensable for disposal of this ERAD-L_M_ substrate.

As shown in **Figs. 1C–1D**, CsA substantially inhibited disposal of BACE457Δ (**Figs. 2D–2E**, lane 3 *vs* lane 2). Consistent with the identification of CyPB as the intracellular target of CsA modulating disposal of this ERAD-L_S_ substrate, the down-regulation of CyPB substantially delayed BACE457Δ disposal (**Figs. 2D–2E**, lane 5 *vs* lane 2). Exposure of cells with low intralumenal content of CyPB to CsA had a minor, additional inhibitory effect on BACE457Δ disposal (lane 6 *vs* lane 5) possibly due to the inhibition of the residual CyPB remaining in the cells subjected to specific RNAi (**Fig. 2A**, lane 2). In contrast, the down-regulation of CyPA did not delay BACE457Δ disposal (lane 8 *vs* lane 2) and CsA maintained the inhibitory effect on BACE457Δ disposal in cells with low levels of CyPA (lane 9). The disposal of CD3δΔ that was insensitive to CsA (**Figs. 1E–1F**) was also not inhibited upon variations in the intracellular levels of CyPB and of CyPA (**Figs. 2F–2G**).

These data are consistent with a selective involvement of the luminal immunophilin CyPB in clearance of ERAD-L_S_ substrates characterized by the presence of *cis* proline residues.

### The enzymatic activity of CyPB is required to regulate ERAD

The data shown so far are consistent with a model in which CyPB facilitates BACE457Δ disposal by assisting the enzymatic conversion of peptidyl-prolyl bonds of the misfolded substrate from the *cis* into the *trans* configuration. This could eliminate turns in the polypeptide chain thus facilitating protein dislocation across the ER membrane, which is required for ERAD and occurs through an elusive proteinaceous channel [16]. Alternatively, peptidyl-prolyl isomerization could facilitate another rate-determining step in the disposal pathway of ERAD-L_S_ substrates, for example their dissociation from a luminal retention factor. To assess whether the catalytic activity of CyPB is required for BACE457Δ disposal, an active and a catalytically inactive CyPB carrying a R62A mutation that substantially reduces the prolyl isomerization activity *in vitro* [30] were back transfected in cells with a reduced content of endogenous CyPB (**Fig. 3A**). Both recombinant CyPB and CyPB_R62A_ carried three silent mutations in their coding sequence to render their transcripts resistant to the small interfering RNA used to down-regulate endogenous CyPB. Ectopic expression of CyPB in cells with reduced level of the endogenous protein re-established efficient disposal of BACE457Δ (**Figs. 3B–3C**, lane 8 *vs* lane 5). In these cells, like in cells with normal content of endogenous CyPB (lanes 1–3), exposure to CsA substantially delayed BACE457Δ disposal (lane 9 *vs* lane 8). In contrast, ectopic expression of the enzymatically inactive CyPB_R62A_ was not sufficient to recover BACE457Δ disposal in cells depleted of the endogenous enzyme (lane 11 *vs* lanes 2 and 8). This indicates that the enzymatic activity is required for CyPB-assisted acceleration of BACE457Δ disposal and implies that enzymatic conversion of one or more of the *cis* peptidyl-prolyl bonds of BACE457Δ facilitates disposal of the terminally misfolded polypeptide. These results are also consistent with the finding that CyPB is dispensable for efficient disposal of CD3δΔ, an ERAD-L_S_ substrate lacking proline residues in the *cis* configuration (**Figs. 1E–1F** and **Figs. 2F–2G**).

### *Cis* proline replacement abrogates CsA-sensitivity and CyPB-dependency of ERAD

BACE457Δ has 24 proline residues. It is impossible to establish which peptidyl-prolyl bond needs to be interconverted from the *cis* into the *trans* configuration during the short retention in the ER lumen that precedes dislocation into the cytosol of this folding-defective polypeptide. However, we determined whether replacement of Pro84, 146 and 390 (which are in *cis* in the stable BACE501 splice variant) with alanine residues relieved the CyPB-dependency for efficient disposal.

**Figure 4:**
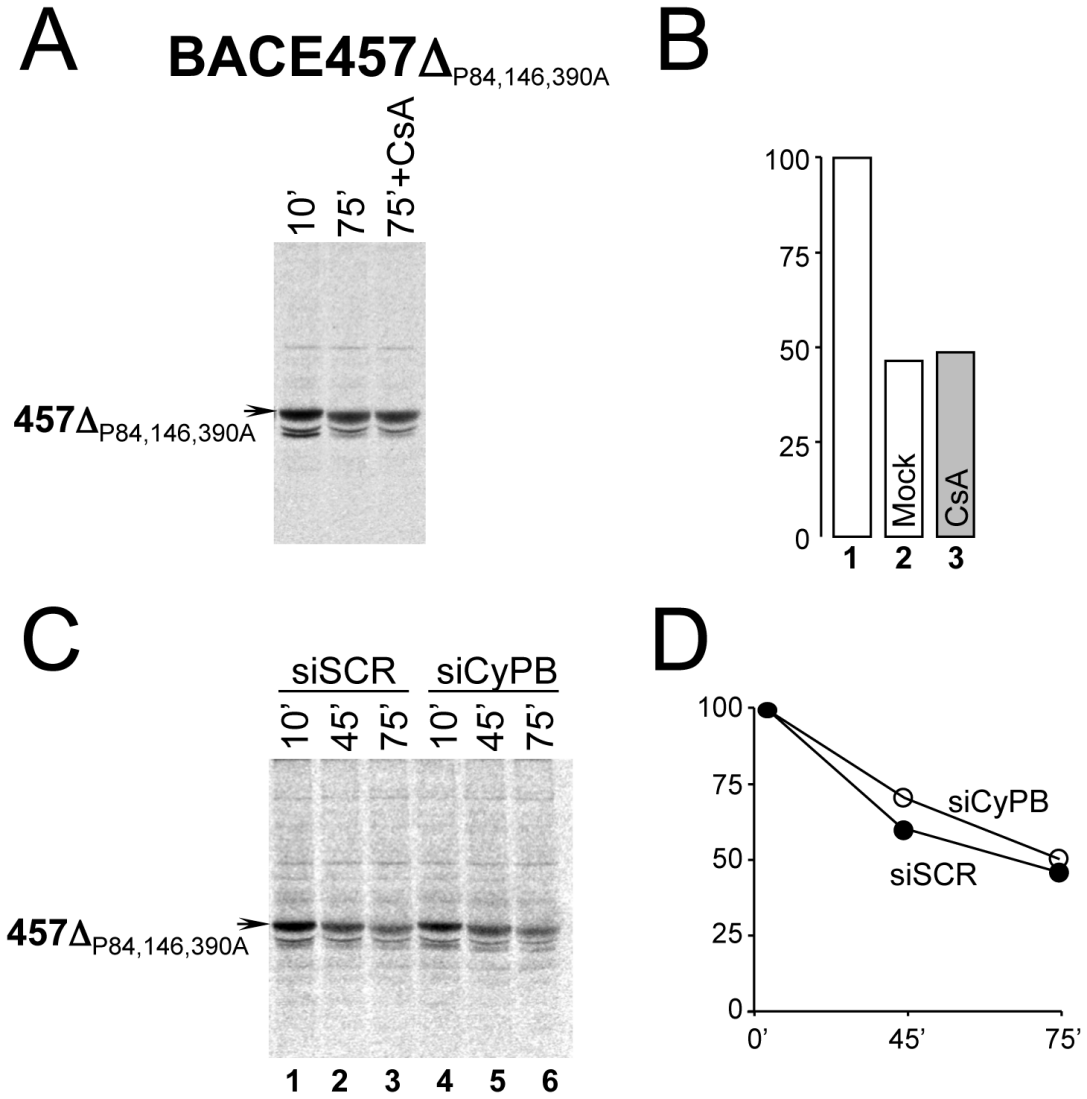
Consequences of CsA-treatment and of CyPB down regulation on disposal of BACE457Δ in which the three *cis* proline have been replaced by alanine residues. **A** Radiolabeled BACE457Δ_P84,146,390A_ was immunoisolated from total cell extracts after 10 min (lane 1) or 75 min of chase (lane 2), or from cells exposed for 75 min to CsA (lane 3). **B** Quantification of the labeled polypeptide bands. **C** Radiolabeled BACE457Δ_ P84,146,390A_ was immunoisolated at the end of the chase times from detergent-extracts of cells expressing a scrambled siRNA (siSCR, lanes 1–3) or a siRNA targeting CyPB (siCyPB, lanes 4–6). **D** Quantification of the labeled polypeptide bands.

Consistent with a CsA-insensitive ERAD pathway for ERAD-L_S_ proteins lacking *cis* proline residues (**Figs. 1E–1F**), disposal of BACE457Δ_P84,146,390A_ was not inhibited by cell incubation with CsA (**Figs. 4A–4B**). Similarly, while reduction in the intralumenal level of endogenous CyPB substantially delayed disposal of the wt BACE457Δ (**Figs. 2** **–** **3**), degradation of BACE457Δ_P84,146,390A_ remained unperturbed upon depletion of the ER-resident immunophilin (**Figs. 4C–4D**, lanes 4–6 *vs* 1–3). Taken together, these data show that the enzymatic activity of CyPB is only required for disposal of non-membrane tethered BACE457Δ containing *cis* peptidyl-prolyl bonds.

All in all, CsA was identified as the first selective inhibitor of disposal of a soluble (ERAD-L_S_), but not of a membrane-tethered (ERAD-L_M_) version of a misfolded polypeptide with luminal structural defects. This confirms that tethering at the ER membrane changes the requirements for efficient polypeptide clearance from the mammalian ER lumen [15]. The CsA-sensitive step of ERAD occurs after substrate de-mannosylation and before intervention of cytosolic proteasomes (both progressing unperturbed in cells exposed to CsA (legend of **Fig. 1C** and [31]). We identify CyPB as the ER-resident target of CsA involved in disposal from the mammalian ER of some (e.g., BACE457Δ, BACE476Δ, NHK) but not all (e.g., CD3δΔ) ERAD-L_S_ substrates. We provide evidence that the intervention of CyPB in ERAD requires a functional active site. As such, our data are the first demonstration of enzymatic intervention of a member of the PPI superfamily in protein quality control in the ER of living cells. We hypothesize that the presence of peptidyl-prolyl bonds in the *cis* configuration is a characteristic of those ERAD-L_S_ substrates that show CsA-sensitive, CyPB-dependent disposal. For these misfolded polypeptides, consequences of CsA exposure or of reduction in the intralumenal level of CyPB are comparable to consequences of inactivation of components of the dislocon complex built around the membrane-embedded E3 ubiquitin ligase HRD1 that are stringently required for disposal of ERAD-L_S_ proteins [15]. Our hypothesis that CyPB participates in the HRD1/ERAD-L_S_ pathway is consistent with a recent report showing that CyPB forms a functional complex with GRP94, another component of the HRD1 pathway [14], to protect cells against ER stress [32]. Finally, our data imply that unfolding of non-native polypeptides upon *cis* to *trans* isomerization of peptidyl-prolyl bonds might facilitate dislocation across the ER membrane [9] similarly to what has been proposed for polypeptide unfolding upon PDI-catalyzed reduction of intra- and inter-molecular disulfide bonds [5]. Alternatively, it could promote dissociation of misfolded polypeptides from ER retention factors thus facilitating dislocation across the ER membrane.

## Materials and Methods

### Expression plasmids, antibodies and inhibitors

Plasmids and antibodies for NHK, CD3δΔ and BACE variants are described in [15], [23]. The plasmid for CyPB expression is described in [33]. Primers for silent mutations that protect ectopic CyPB from siRNA (CyPB, 5′-AAAGA CTGTTCCAAAAACCGTAGACAATTTTGTGGCCTTAGCT-3′). Primers for generation of inactive CyPB_R62A_ (5′-GGCTACAAAAACAGCAAATTCCATGCTGTAAT CAAGGACTTCATG-3′). Primers for generation of BACE457Δ_P84,146,390A_, which lacks *cis* prolines (5′-CCGTGGGCAG GCCCCGCAGACG-3′, 5′-GGCACCGACCTGGCTGACGAC TCCC-3′, 5′-CAGCGGTGGAAGGCGCTTTTGTCACCTTG-3′). Mutants were generated using the site-directed mutagenesis kit (Stratagene). DNA preparations were obtained using commercially available purification kits (Sigma). The nucleotide sequences of all plasmids were verified on both strands. Antibodies against CyPB, CyPA and Tubulin were from ABR, Biomol and ABM. The proteasome inhibitor PS341 was a kind gift of Millenium Pharmaceuticals Inc and was used at a concentration of 9 µM. Kifunensine (Toronto Research Chemicals Inc), thapsigargin (Sigma) and CsA (Bedford Labs) were used at a concentration of 100 µM, 300 nM and 20 µM, respectively. All inhibitors were only included in the chase media.

### Cell Lines, transient transfections, RNA interferences, metabolic labelling, immunoprecipitations, immunoblots and analysis of data

HeLa cells (from ATCC) were grown in MEM Alpha supplemented with 10% FBS. Cells at 80–90% confluence in a 6 cm tissue culture plate were transfected with the expression plasmid of interest (4 µg for single transfections, 6 µg total DNA for double transfections) using Lipofectamine2000 (Invitrogen) according to the manufacturer instructions. Experiments were normally performed 17 hours after transfection. For siRNA-based interference, HeLa cells at 50% confluence in a 3.5 tissue culture plate were transfected with siRNA duplex (Ambion Inc, 50 pmol/dish) using Lipofectamine2000 according to the manufacturer instructions. Four hours after transfection, the medium was replaced with MEM Alpha supplemented with 1% of non-essential amino acids (GIBCO). Thirty hours after siRNA transfection, cells were transfected with the expression plasmids of interest. Experiments were performed 48 hours post-siRNA transfection. siRNA targeting sequences: CyPB: CAAAAACAGUGGAUAAUUU; CyPA: CUGGAUUGCAGAGUUAAGU.

Seventeen hours after transfection, cells were starved for 20 min in Met/Cys free medium, pulsed for 10 min with 50 µCi [S^35^]Met/Cys and chased for the indicated times with MEM Alpha supplemented with 5 mM cold Met/Cys. Postnuclear supernatants (PNS) were prepared by solubilization of cells in 400 µl/3,5 cm dish (or 800 µl/6 cm dish) ice-cold 2% CHAPS (Anatrace) in HEPES-buffered saline (HBS), pH 6.8 containing 20 mM N-ethylmaleimide and protease inhibitors. CHAPS-insoluble material was separated by centrifugation at 10’000 g for 10 min. Immunoprecipitations were performed by adding protein A beads (Sigma; 1∶10, w/v swollen in HBS) with the selected antibody for 2h at 4°C. Immunoprecipitates were extensively washed (3×10 min) with 0.5% CHAPS in HBS, resuspended in sample buffer, boiled for 5 min and finally separated in SDS-PAGE. Gels were exposed to BioMax (Kodak) films and scanned with an Agfa scanner. Relevant bands were quantified by ImageQuant software (Molecular Dynamics). Immunoblots were performed using the SNAP i.d. protein detection system (Millipore). All primary antibodies were used at 1∶200–1∶333 dilutions. Secondary antibodies were HRP-conjugated and used at 1∶10’000 dilutions. The ECL-Plus detection system was from Amersham.

### Proline residues in the *cis* conformation

Identification of proline residues in the *cis* conformation was done by using the WHAT IF Wb Interface (http://swift.cmbi.ru.nl/servers/html/index.html) [34].
